# Inhibitory Effect of Fluralaner on GABA Receptor Subunit RDL of *Bactrocera dorsalis*

**DOI:** 10.3390/insects16050479

**Published:** 2025-05-01

**Authors:** Xiangyi Zhou, Guoxing Chen, Keying Chen, Zhanyi Xu, Jiali Qian, Ru Yan, Bosheng Chen, Huiming Wu, Mengli Chen

**Affiliations:** 1 Zhejiang Key Laboratory of Biology and Ecological Regulation of Crop Pathogens and Insects, College of Advanced Agricultural Sciences, Zhejiang A&F University, Hangzhou 311300, China; 17767273866@163.com (X.Z.); guoxingchen@stu.zafu.edu.cn (G.C.); keying_chen02@163.com (K.C.); bchen13@zafu.edu.cn (B.C.); wuhm@zafu.edu.cn (H.W.); 2 Institute of Pesticide and Environmental Toxicology, College of Agriculture and Biotechnology, Zhejiang University, Hangzhou 310030, China; 20156820@zju.edu.cn (Z.X.); jialiqian@zju.edu.cn (J.Q.); 3 College of Life Sciences, Zhejiang University, Hangzhou 310030, China; yanru@zju.edu.cn

**Keywords:** *Bactrocera dorsalis*, RDL, fluralaner, electrophysiology

## Abstract

*Bactrocera dorsalis* (Hendel) (Diptera: Tephritidae), a highly destructive pest, affects fruits and vegetables globally, leading to significant economic losses. As a homomeric subunit of γ-aminobutyric acid (GABA) receptors, RDL plays a crucial role in various physiological activities in insects. However, functional characterization of RDL from *B. dorsalis* has not yet been elucidated. Here, we cloned the *Rdl* gene from *B. dorsalis*, *BdRdl*, and investigated the expression pattern of *BdRdl*. The results showed that *BdRdl* was highly expressed in pupae and the heads of male and female adults. Also, we characterized the BdRDL in *Xenopus oocytes* through two-electrode voltage clamping. It turned out that the inward current of BdRDL induced by GABA was followed in a dose-dependent manner with a median effective concentration (EC_50_) of 2.4 × 10^−4^ M. Additionally, we determined the mode action of fluralaner, a new insecticide, on BdRDL in oocytes. We found that fluralaner significantly inhibited the currents induced by GABA, suggesting that fluralaner worked as an antagonist of BdRDL. Furthermore, we found that fluralaner exhibited a comparable insecticidal activity to avermectin against *B. dorsalis* adults. Lastly, the modeling and molecular docking predicted that fluralaner interacted with RDL via hydrogen bonds. Our results not only characterized the RDL of *B. dorsalis*, but also revealed that fluralaner works as an antagonist of BdRDL and could be used as an effective strategy for *B. dorsalis* control.

## Simple Summary

*Bactrocera dorsalis* (Hendel) (Diptera: Tephritidae), a highly invasive pest with high transmissibility and adaptability, causes severe damage to various commercial vegetables and fruits. RDL, as the homomeric subunit of γ-aminobutyric acid (GABA) receptors, is the target of several insecticides. In this study, we found that *BdRdl* was highly expressed in the pupae and heads of male and female adult insects. Also, BdRDL was functionally characterized in *Xenopus oocytes* by two-electrode voltage clamping and we found that fluralaner worked as an antagonist of BdRDL. Furthermore, we found that fluralaner exhibited a comparable insecticidal activity to avermectin against *B. dorsalis* adults. Finally, the modeling and molecular docking predicted that fluralaner interacted with BdRDL via hydrogen bonds. These results suggested that fluralaner, as an antagonist of BdRDL, could be used to control *B. dorsalis*.

## 1. Introduction

The Oriental fruit fly, *Bactrocera dorsalis* (Hendel) (Diptera: Tephritidae), is a serious invasive pest with high transmissibility and adaptability [1,2]. *B. dorsalis* is highly polyphagous and causes severe damage to over 250 species of commercial vegetables and fruits, such as oranges, apples, pumpkins and cucumbers [3,4]. Currently, the management of *B. dorsalis* heavily relies on the application of insecticides; however, overuse of pesticides leads to the development of greater resistance to insecticides [5]. Therefore, it is urgent to develop new insecticides to manage *B. dorsalis*.

Ligand-gated ion channels (LGICs), a group of transmembrane ion channel proteins, mediate rapid excitatory and inhibitory synaptic transmission in the insect nervous system, including nicotinic acetylcholine (nACh) receptors, 5-hydroxytryptamine (5-HT) receptors, glutamate-gated chloride (GluCl) channels and γ-aminobutyric acid (GABA) receptors [6]. RDL (resistant to dieldrin), one subunit of GABA receptors, is able to form functional pentameric ligand-gated ion channels. RDL consists of a long N-terminal extracellular domain and four transmembrane regions (TM1-TM4), of which TM2 is integral in forming the intact chloride channel [7,8]. Numerous studies have shown that RDL is an important target for various insecticides [7,9,10], such as cyclodienes, phenylpyrazoles, macrocyclic lactones, meta-diamides and isooxazoline [7,11].

Fluralaner, a novel isoxazoline-class compound, used widely to treat flea and tick infections in dogs and cats; however, in recent years, fluralaner has been used as a highly effective insecticide and acaricide in the control of sanitary and agricultural pests [12]. Previous studies reported that fluralaner exhibited high toxicity against many pests, including *Musca domestica*, *Chilo suppressalis*, *Tribolium castaneum*, *Phyllotreta striolata*, *Aedes aegypti* and *Solenopsis invicta* [13,14,15,16,17]. Fluralaner exerts its insecticidal activity by selectively binding to the GABA-gated chloride channels in the nervous systems of arthropods. Specifically, it inhibits the function of the GABA receptor, particularly the RDL subunit [18,19,20]. The disruption on GABA leads to hyperex-citation, paralysis and the eventual death of the target pests.

So far, RDL subunits from many insect species have been characterized in *Xenopus oocytes*, including *A. aegypti*, *P. xylostella*, *Varroa destructor*, *Apis mellifera*, *C. suppressalis* and *Drosophila melanogaster* [6,11,21,22,23]. However, the RDL of *B. dorsalis* has not yet been elucidated. In this study, we cloned the *Rdl* gene from *B. dorsalis* (*BdRdl*), and verified the expression patterns of BdRdl in different developmental stages and tissues. Next, we functionally characterized the BdRDL in *Xenopus oocytes*, and identified the molecular mechanism of fluralaner on BdRDL. In addition, we explored the sensitivity of *B. dorsalis* to fluralaner. Lastly, the interaction between BdRDL and fluralaner was predicted through modeling and molecular docking. Our results not only elucidate the molecular mechanism of fluralaner on BdRDL, but also provide a theoretical basis for the in-depth study of the development of insecticides for the management of *B. dorsalis*.

## 2. Materials and Methods

### 2.1. Insects and Chemicals

The sensitive-strain *B. dorsalis*, kindly provided by Dr. Liwei Meng from Southwest University, was maintained at 25 ± 1 °C under 60–80% relative humidity (RH) with a photoperiod of 14:10 (L:D). GABA was purchased from Yuanye Bio-Technology (Shanghai, China). The 99% Avermectin mixture and 99% fluralaner were purchased from Aladdin (Shanghai, China).

### 2.2. Bioassay

A topical bioassay was used to examine the toxicity of avermectin and fluralaner to *B. dorsalis* adults. Avermectin and fluralaner were separately dissolved in acetone and serially diluted to specific required concentrations. Twenty 5- to 10-day-old *B. dorsalis* adults were anesthetized with CO_2_ and treated with 2 µL of insecticide solution using a microsyringe (Hamilton PB-600-1, Bienne, Switzerland). After treatment, flies were reared in a glass bottle (6.7 × 9.2cm, 240 mL) with a cotton ball stained with 10% sucrose solution. Mortality was recorded 24 h after treatment. Flies were regarded as dead if they could not move after being gently stimulated with a small paintbrush. Solvent acetone was served as a control. Three replicates were conducted for each chemical dilution. Statistical analyses were performed using the probit procedure from the DPS software (Version 7.05).

### 2.3. Cloning and Sequencing Analysis of BdRdl

Total RNA was extracted from the excised heads of *B. dorsalis* adults using RNAiso Plus (TaKaRa, Dalian, China), following the manufacturer’s instructions. RNA quality was checked by agarose electrophoresis and spectrophotometer (Allsheng, Hangzhou, China). cDNA was synthesized with a SuperScript^®^ III First-Strand Synthesis kit (Invitrogen, Carlsbad, CA, USA) according to the manufacturer’s instructions. The specific primers were designed by Beacon Designer 8.0 (Premier Biosoft, Palo Alto) based on the Open Reading Frame of *BdRdl* (GenBank accession number: XM_049458762) (Table 1). The PCR product was generated using Phusion^®^ High-Fidelity DNA Polymerase (New England BioLabs, Massachusetts, MA, USA) and cloned into the pEASY-Blunt Zero cloning vector (Transgen BioTech, Beijing, China). The method used to create the phylogenetic tree was identical to those previously described [24]. The RDL sequences from different species were aligned using MAFFT v.7 with the auto-option strategy. The maximum-likelihood tree was generated with IQ-TREE, and LG + G8 + F was selected as the best-fit model according to the Bayesian information criterion. Bootstrap support values were based on 1000 replicates. The tree was visualized using iTOL (https://itol.embl.de) (Accessed on 6 February 2025).

### 2.4. Relative Expression Pattern of BdRdl

To identify the expression pattern of *BdRdl*, the relative transcript levels of different stages and tissues were examined by RT-qPCR. RNA was extracted from flies at different development stages, including eggs, larvae and adults, and different tissues, including heads, legs, thoraxes and abdomens, using RNAiso Plus (TaKaRa, Dalian, China). The cDNAs were synthesized using the PrimeScript RT reagent Kit with gDNA Eraser according to the manufacturer’s instructions (TaKaRa, Dalian, China). The *EF1a* and *Tublin* were used as reference genes. Primers of *EF1a*, *Tublin* and *BdRdl* were designed using Beacon Designer 8.0 (Premier Biosoft, Palo Alto) (Table 1) and the primer amplification efficiencies were verified as shown in Figure S1. The RT-qPCR was performed using TB Green^®^ Premix Ex Taq™ II (Tli RNaseH Plus) (TaKaRa, Dalian, China) on QuantStudio3 Real-Time PCR System (Ap-plied Biosystems, Foster City, CA, USA). Three independent biological replicates and three technical replicates were conducted. The relative expression levels of *BdRdl* in the different stages and tissues were calculated using the 2^−∆∆Ct^ method.

### 2.5. Characterization of BdRDL in Xenopus oocytes

The specific primers of *BdRdl* containing the T7 promoter are shown in Table 1. The PCR products were subcloned into the PGH19 vector with the In-Fusion HD cloning kit (TaKaRa Bio, Otsu, Japan). The PGH19 vector was kindly provided by Prof. Ke Dong from Duke University. The recombinant plasmid was verified by sequencing and used for cRNA preparation. cRNA was prepared by in vitro transcription with T7 polymerase using the mMESSAGE mMACHINE^®^ high yield capped RNA kit (Ambion, Austin, TX, USA). Ovarian lobes were isolated from female *Xenopus laevis* frogs and the procedures for oocyte preparation and injection were identical to those described previously [24]. Each oocyte was injected with 15.6 ng *BdRdl* cRNA and incubated at 18 °C to examine the properties of BdRDL.

Electrophysiological recording was conducted by using the oocyte clamp instrument OC725D (Warner Instrument, Hamden, CT, USA) and Digidata 1550B (Axon Instruments Inc., Foster City, CA, USA). A stock solution of GABA was prepared with sterile water, and a stock solution of fluralaner was prepared with dimethyl sulfoxide (DMSO). The working solution was prepared in an ND96 recording solution immediately prior to the experiment. The final concentration of DMSO in the working solution was less than 0.1% (*v*/*v*). The oocytes were clamped at −60 mV and the flow rate of the working solution was 2 mL/min in the perfusion delivery system. For the agonist GABA, the perfusion time was 20 s. For the antagonist fluralaner, the oocytes were first stimulated with GABA (EC_50_), followed by perfusing fluralaner alone for 2 min, and then GABA (EC_50_) was co-applied with fluralaner for 20 s. The concentration–response curves were normalized and fitted with the Hill equation.

### 2.6. Homology Modeling and Molecular Docking

AlphaFold3 (https://alphafold.ebi.ac.uk/) was applied to model the three-dimensional (3D) structure of BdRDL (accessed on 16 January 2025). The ligand structure was obtained from the PubChem database (https://pubchem.ncbi.nlm.nih.gov/) (accessed on 16 January 2025). The non-covalent interactions between the proteins and compounds were analyzed using the Maestro suite in the Schrödinger software (version 13.7). The interactions between protein and ligands were visualized using PyMol 2.5 and the Protein–Ligand Interaction Profiler.

## 3. Results

### 3.1. BdRdl Sequence Analysis

**Figure 1:**
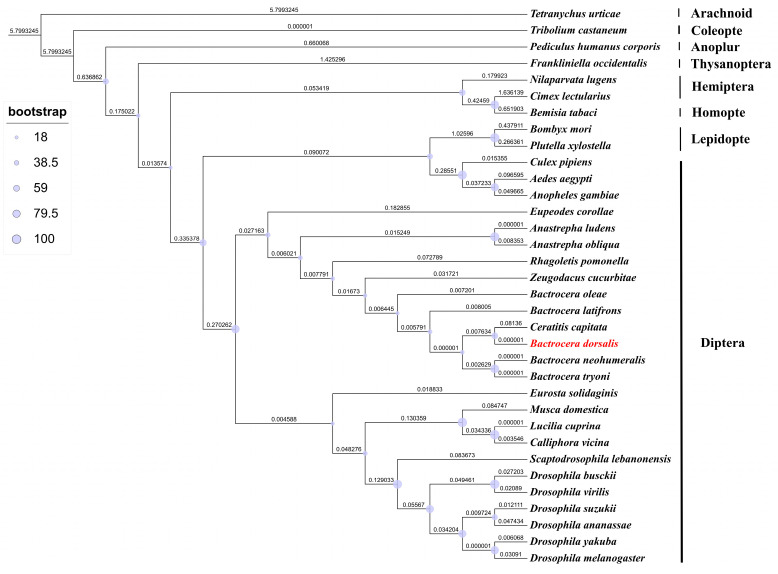
Phylogenetic tree of RDL ortholog sequences from multiple insect species.

**Figure 2:**
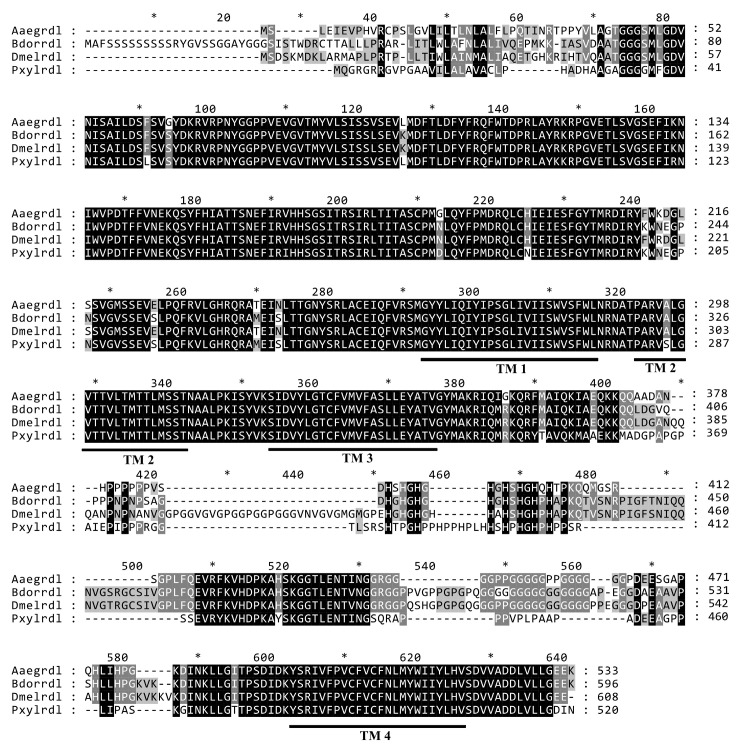
The alignment of amino acid sequences between *Drosophila melanogaster*, *Aedes aegypti*, *Plutella xylostella* and *Bactrocera dorsalis*. The transmembrane regions are marked as TM1-TM4.

The ORF of the *BdRdl* consists of 1788 bp and encodes a protein consisting of 596 amino acids. A phylogenetic tree was generated using RDL proteins from a total of 34 species of different orders such as *D. melanogaster*, *A. aegypti*, *Bombyx mori* and so on (Figure 1). Furthermore, the amino acid sequences of RDL from *D. melanogaster*, *A. aegypti*, *P. xylostella* and *B. dorsalis* were aligned, and it turned out that all RDLs contained four transmembrane regions (TM1-TM4), which were highly conserved in these species (Figure 2).

### 3.2. Expression Profiles of BdRdl

**Figure 3:**
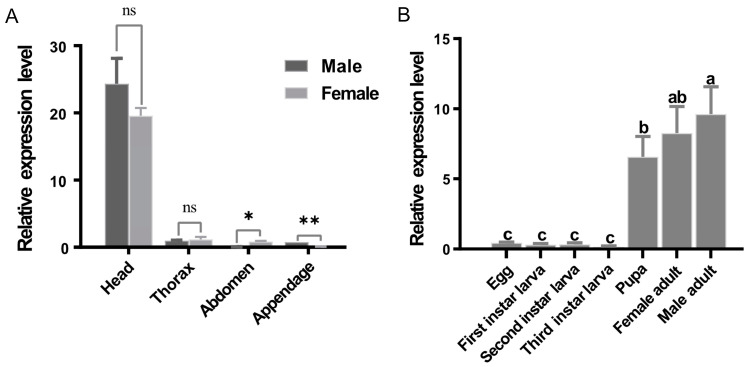
The expression patterns of *BdRdl*. (**A**) Transcript levels of *BdRdl* in different male or female tissues. ns indicate no significant, * and ** indicate significant difference among female and male tissues at the *p* < 0.05 level and *p* < 0.01 level followed by Student’s *t*-test. ns indicates no significant difference. (**B**) Transcript levels of *BdRdl* in different developmental stages. Data are plotted as mean ± SEM. Different letters above the bars indicate significant difference among different stages at the *p* < 0.05 level followed by Tukey’s honest significant difference test.

We used RT-qPCR to examine the expression patterns of *BdRdl* in different developmental stages and different tissues of male or female *B. dorsalis* adults. The results showed that *BdRdl* was biasedly expressed in the head of *B. dorsalis* and was rarely expressed in other parts, including the thorax, abdomen and appendages (Figure 3A). In addition, we found that *BdRdl* was rarely expressed in eggs or larvae, and transcription level of *BdRdl* was increased in pupae and both male and female adults (Figure 3B).

### 3.3. Functional Characterization of BdRDL in Xenopus oocytes

**Figure 4:**
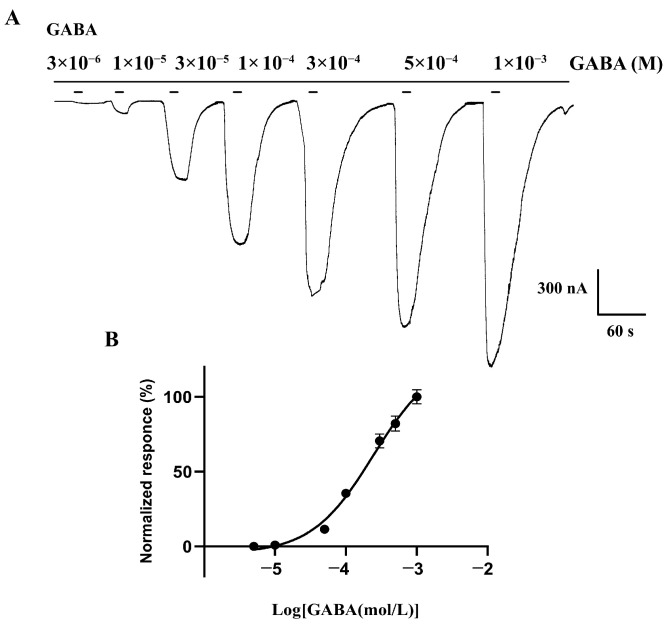
GABA-induced currents in BdRDL. (**A**) Representative currents induced by GABA from BdRDL expressed in oocytes. (**B**) Concentration response curves of GABA. Data are plotted as mean ± SEM; n = 6.

To characterize the function of BdRDL, the cRNA of BdRDL was injected into the *Xenopus* oocytes, and two-electrode voltage clamping was used to examine the properties and sensitivity of BdRDL against the agonist GABA. The homomeric receptor with ion channels was successfully expressed in *Xenopus* oocytes and the current was generated by GABA. As shown in Figure 4, the current induced by GABA was followed in a dose-dependent manner. The maximum current was induced by 1 mM GABA and the EC_50_ of GABA was 2.4 × 10^−4^ M.

### 3.4. Inhibition Effects of Fluralaner BdRDL in Xenopus oocytes

**Figure 5:**
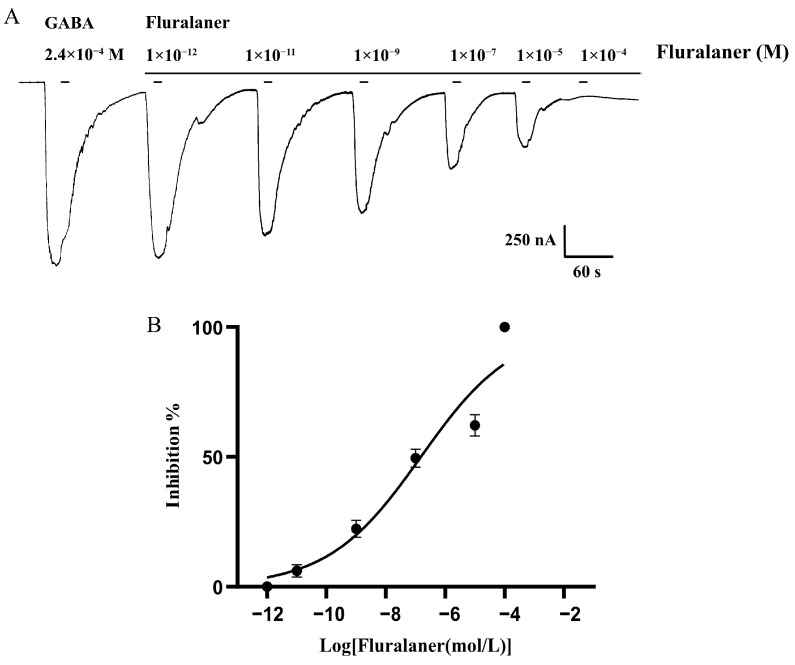
The inhibition effects of fluralaner on BdRDL. (**A**) Representative currents induced by 2.4 × 10^−4^ M GABA from BdRDL in the presence of fluralaner. (**B**) Fluralaner inhibition of 2.4 × 10^−4^ M GABA-induced currents from BdRDL. Data are plotted as mean ± SEM, n = 6.

We found that no currents were generated when the simulation was carried out with fluralaner alone, and fluralaner significantly inhibited the currents induced by GABA, indicating that fluralaner was an antagonist of BdRDL. To evaluate the inhibitory effects of fluralaner, the antagonistic effects of serial dilutions on RDL induced by GABA at dose of EC_50_ were examined. The results showed the inhibitory effects were followed in a dose-dependent manner, and the inhibition percentage was increased with an increase in the concentration of fluralaner (Figure 5). The IC_50_ value of fluralaner was 1.5 × 10^−7^ M.

### 3.5. Sensitivity of B. dorsalis Adults to Fluralaner

A topical bioassay was used to determine the sensitivity of *B. dorsalis* adults to fluralaner. To better evaluate the toxicity of fluralaner, we used avermectin, one of the macrocyclic lactone insecticides, as a positive control. In the assay, no *B. dorsalis* died when they were treated with the solvent control. Both fluralaner and avermectin had distinct insecticidal activity against *B. dorsalis* adults. The LC_50_ of fluralaner and avermectin were 8.281 mg/L and 4.307 mg/L, respectively (Table 2).

### 3.6. Modeling of Ligand Binding to BdRDL

**Figure 6:**
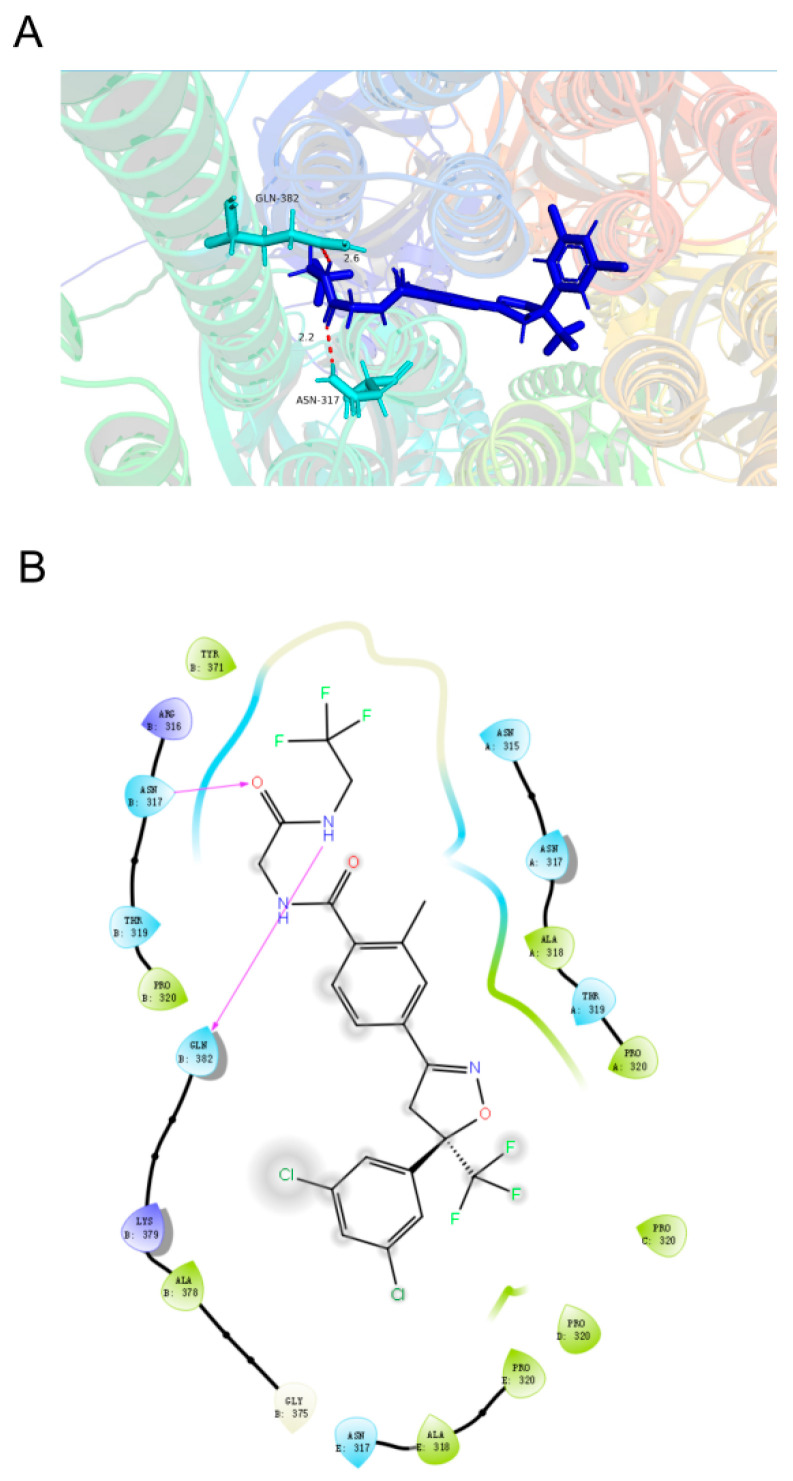
The predicted binding modes of fluralaner targeting BdRDL. The top view of the complex structures (**A**) and two-dimensional ligand interaction diagrams (**B**) show the binding modes of fluralaner in BdRDL. The rainbow colors indicated the helix structures of different subunits of BdRDL.

To better understand the molecular mechanism of fluralaner on RDL, we predicted the interaction between BdRDL and fluralaner through modeling and docking using AlphaFold3. The BdRDL subunit clearly formed a functional pentameric ion channel in vivo, and the key binding site of fluralaner was located on the transmembrane domain (Figure 6). The molecular docking predicted that fluralaner interacted with Asn 317 via a hydrogen bond of 2.2 Å length and with Gln 382 via a hydrogen bond of 2.6 Å length (Figure 6A,B).

## 4. Discussion

Fluralaner, a novel insecticide, has been used in veterinary medicine for the treatment of fleas, ticks and other parasites in animals such as dogs, cats, poultry and cattle [12]. Recently, numerous studies have reported that fluralaner also exhibits toxicity against many agricultural and sanitary pests [17,25,26]. A previous study even reported that fluralaner showed a higher toxicity towards *Spodoptera litura* than fipronil and chlorpyrifos [27]. Liu et al. (2021) argued that fluralaner could be used as an effective pesticide against vegetable pests and would not affect the life history traits of *Propylaea japonica* at a sublethal level, indicating fluralaner is suitable to be applied in greenhouses and open fields [14]. Here, we determined the high toxicity of fluralaner against *B. dorsalis* adults through a topical assay. We found that fluralaner exhibited a comparable toxicity towards *B. dorsalis* adults as avermectin, a widely used insecticide for controlling agricultural and horticultural pests. Our results were consistent with previously published results showing that fluralaner exhibited strong lethal effects against three fruit fly species, *B. dorsalis*, *Bactrocera cucurbitae* and *Bactrocera tau* [28]. These results demonstrated that fluralaner has great potential for controlling *B. dorsalis*.

RDL receptors are widely expressed throughout insects’ central nervous system (CNS) and play a crucial role in inhibitory neurotransmission [8]. Consistent with previous reported by Wang et al. [6], we found that *Rdl* was highly expressed in the head of male and female adults, and rarely expressed in other tissues. Different from *Rdl* genes in lepidopteran insects, such as *P. xylostella*, *B. mori* and *C. suppressalis* [17,21], only one *Rdl* gene was found in *B. dorsalis*, as in many insects, such as *M. domestica*, *D. melanogaster* and *A. mellifera* [29,30,31].

In accordance with RDLs from other insects, the BdRDL subunit alone forms the functional homotetramer in the oocytes, and GABA induced dose-dependent currents. The EC_50_ of GABA was 2.4 × 10^−4^ M, which was similar to those values reported for the RDLs of *A. aegypti*, *P. xylostella* and *M. domestica* [6,11,21,22,29]. Wang et al. (2022) reported that 2.5 μM ivermectin, a semi-synthetic derivative of avermectin, induced weak inward currents on the RDL of *A. aegypti* without the participation of GABA; however, fluralaner alone did not induce any weak inward currents [6]. Consistent with the results described, we found that fluralaner alone could not activate BdRDL, and fluralaner exhibited an inhibitory effect on BdRDL. Many studies have shown that avermectin simultaneously inhibits, activates and enhances the inward current in the RDL of *Anopheles gambiae*, human louse *Pediculus* and *Tetranychus cinnabarinus* [21,32,33]. These results suggest that fluralaner and ivermectin or avermectin might have a distinct mode of action on the RDL. Except for targeting the RDL, as an antagonist of chloride channels, avermectin causes changes in the permeability of γ-aminobutyric acid (GABA)-gated chloride channels (GABACls), L-glutamate-gated chloride channels (GluCls), histamine-gated chloride channels and pH-sensitive chloride channels, resulting in neurotransmission disruptions that lead to hyperpolarization, paralysis and the eventual death of neuronal membranes [34,35,36,37,38,39,40]. The previously published papers and our study indicated that fluralaner inhibited the function of RDL, whereas the other target sites of fluralaner, such as GABA-gated chloride channels (GABA-Cl), need to be studied further.

The electrophysiological data showed that the IC_50_ value of fluralaner was 1.5 × 10^−7^ M, which was similar to those values published for the RDL of *A. aegypti* [6]. However, the IC_50_ value of fluralaner was approximately 10- or 100-fold lower in the RDLs of *C. suppressalis*, *Laodelphax striatellus*, *D. melanogaster Tetranychus urticae* and *Apis mellifera* [11,23], suggesting that our BdRDL was less sensitive to fluralaner. A discrepancy between the binding affinities of RDL and the insecticides was common, as shown in *P. xylostella*, PxRDL1 and PxRDL2, which exhibited distinct sensitivities to fipronil in *Xenopus* oocytes. PxRDL2 receptors were 40 times less sensitive to fipronil than PxRDL1 [21]. Such a situation was also found for the RDL of *V. destructor*; one type of VdRDL was nearly 500-fold less sensitive to fipronil or picrotoxin than the other two types of VdRDL [41].

Although both fipronil and fluralaner target insect RDLs, the binding sites of fipronil and fluralaner in the RDL are located independently [42]. Similar to a previous study [23], we predicted that fluralaner would be bound in the helixes of the transmembrane domain of the RDL and interact with RDL via hydrogen bonds. Kono et al. (2022) explored whether fluralaner inhibited the GABA response in the RDL-activated state or resting state, and found that fluralaner might reach the binding site of the activated conformation of RDL in a stepwise fashion and tightly bind to it [19]. Recently, two papers reported that N318L in *D. melanogaster’s* RDL (also known as N316L in *M. domestica*) was identified as a molecular binding site for fluralaner [43,44]. However, the exact interaction between RDL and fluralaner needs to be illuminated through the elucidation of the high-resolution cryo-electron microscopy structures of RDL in either fluralaner-bound or -unbound states.

Although no studies have reported that *B. dorsalis* exhibits resistance to fluralaner, with frequent application, fluralaner resistance will develop soon, as described in previous studies for a strain with >11,000-fold resistance was established in *M. domestica* in four generations, and the cytochrome P450-mediated detoxification and decreased cuticular penetration were two major mechanisms of fluralaner resistance [15,45]. Additionally, the amino acid mutations of RDL were also conferred resistance, such as the mutations N318L and G3’M (TMD3) [11,43]. The elucidation of the molecular mechanism of fluralaner would be helpful to guide the application of fluralaner; for example, the application of different insecticides with distinct modes of action may reduce the risk of resistance developing.

## 5. Conclusions

In conclusion, our study cloned and functionally characterized the BdRDL in oocytes, and showed that fluralaner works as an antagonist targeting BdRDL. Additionally, fluralaner, as well as avermectin, exhibited excellent insecticidal activity against *B. dorsalis*. Our results not only show the great potential of fluralaner in the management of *B. dorsalis*, but also provide more information about the molecular mechanism of fluralaner.

## Figures and Tables

**Table 1:** The primers used in this study.

Primer Name	Primer Sequences (5′–3′)
For RT-qPCR	
*BdorEF1a-*F	CGTTGGTGTCAACAAGATGG
*BdorEF1a-*R	TGCCTTCAGCATTACCTTCC
*BdorTublin*-F	CGCATTCATGGTTGATAACG
*BdorTublin*-R	GGGCACCAAGTTAGTCTGGA
*BdorRdl*qPCR-F	GTGTTCATCATTCTGGAT
*BdorRdl*qPCR-R	ATGTCACGCACTTGTATAG
For amplification	
*BdorRdl*-F	GCCCCTCGTTATTGGTTATAT
*BdorRdl-R*	TAAGCTTGTATGCCAACTGTT
PGH19-F	AATTCTCTAGAGCAAGCTTGATC
PGH19-R	CGGATCCCCGGGGAATTGATCTG
*BdorRdl*PGH19-F	ATCAATTCCCCGGGGATCCGGCCACCATGGCCTTTAGCAGCAGCAG
*BdorRdl*PGH19-R	CAAGCTTGCTCTAGAGAATTTTACTTCTCCTCGCCGAGCAA

Note: The underlined sequences are the homologous sequences of the PGH19 plasmid; the sequence marked by the wavy line is the Kozak sequence.

**Table 2:** Susceptibility of *B. dorsalis* adults to fluralaner and avermectin.

Compounds	Regression Equation	R^2^	LC_50_ (95%CI) (mg/L) ^a^	*p*-Value ^b^	χ2 (df)
Fluralaner	y = 4.8267x − 4.8267	0.9989	8.281 (6.341–10.775)	0.562	2.975 (4)
Avermectin	y = 3.2056x − 2.0328	0.9869	4.307 (3.042–6.760)	0.464	2.563 (3)

^a^ CL: 95% confidence limit. ^b^ *p*-values based on the Chi-square goodness of fit test. *p*-values > 0.5 suggest goodness of fit of the model.

## Data Availability

The data presented in this study are available upon request from the corresponding author.

## Supplementary Materials

The following supporting information can be downloaded at https://www.mdpi.com/article/10.3390/insects16050479/s1, Figure S1: The primer efficiencies for gene *BdEF1a*, *BdTublin* and *BdRDL*.
