# Supplementary figures and images for: Inhibitory Effect of Fluralaner on GABA Receptor Subunit RDL of Bactrocera dorsalis
Source: Insects. 2025 May 1;16(5):479. doi: 10.3390/insects16050479 (PMC12112586; doi:10.3390/insects16050479)

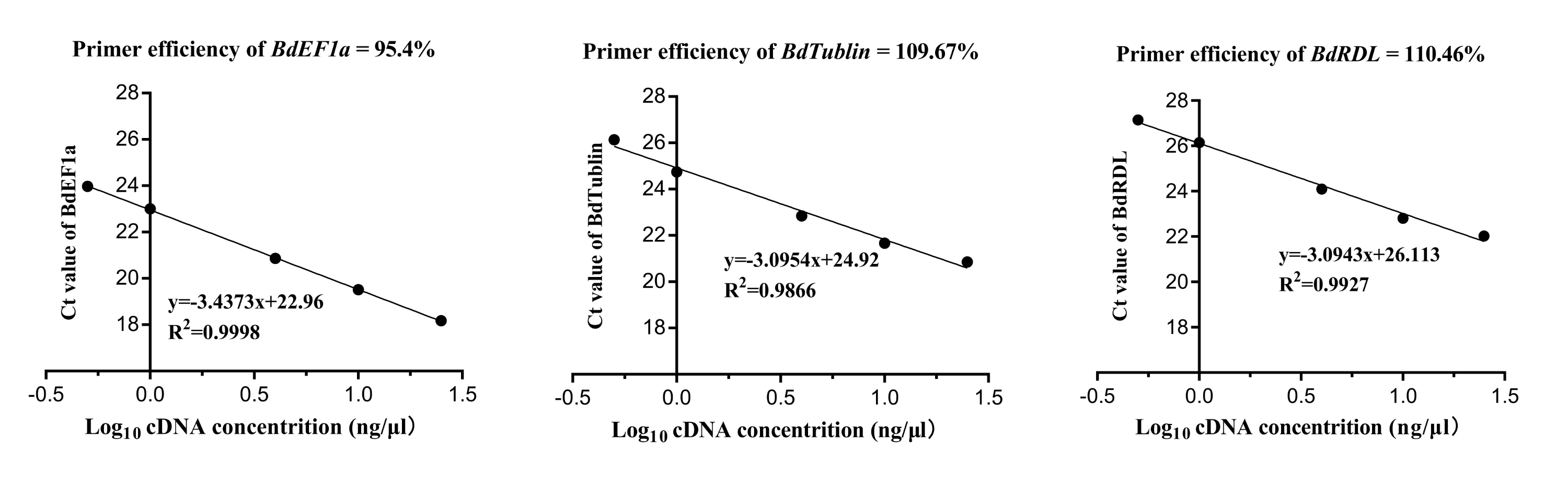

Supplement: Supplementary file 1 [file insects-16-00479-s001.zip › insects-3548171-supplementary.tiff]
